# DV-Curve Representation of Protein Sequences and Its Application

**DOI:** 10.1155/2014/203871

**Published:** 2014-05-08

**Authors:** Wei Deng, Yihui Luan

**Affiliations:** ^1^School of Mathematics, Shandong University, Jinan 250100, China; ^2^School of Science, Shandong Jianzhu University, Jinan 250101, China

## Abstract

Based on the detailed hydrophobic-hydrophilic(HP) model of amino acids, we propose dual-vector curve (DV-curve) representation of protein sequences, which uses two vectors to represent one alphabet of protein sequences. This graphical representation not only avoids degeneracy, but also has good visualization no matter how long these sequences are, and can reflect the length of protein sequence. Then we transform the 2D-graphical representation into a numerical characterization that can facilitate quantitative comparison of protein sequences. The utility of this approach is illustrated by two examples: one is similarity/dissimilarity comparison among different ND6 protein sequences based on their DV-curve figures the other is the phylogenetic analysis among coronaviruses based on their spike proteins.

## 1. Introduction

The graphical representation method has become very common to analyze the huge amount of gene data. Generally, with this method we can first observe visual qualitative inspection in order to recognize major differences among similar gene sequences and further draw some mathematical characterizations of sequences to analyze their similarity/dissimilarity and evolutionary homology.

Letter sequence representation (LSR) of DNA sequences represents each base by a letter of four different letters such as A, T, G, and C. DNA sequences can be represented in different dimension spaces. For example, G-curve and H-curve [1] were first proposed by Hamori and Ruskin before thirty years. Later, Gates [2] established a 2D graphical representation that was simpler than H curve. However, Gate's graphical representation has high degeneracy because of some circuits appearing in its curve. Several researchers in their recent studies have outlined different kinds of DNA sequences graphical representation based on 2D [3–11], 3D [12–15], 4D [16], 5D [17], and 6D [18]. Among these methods, we here stress DV-curve representation which was proposed by Zhang [10]. DV-curve uses two vectors to represent one alphabet of DNA sequences and avoids degeneracy and loss of information. Furthermore, DV-curve has good visualization no matter how long these sequences are and can reflect the length of the DNA sequence.

LSR of protein sequences represents each amino acid by a letter of twenty different letters such as A, R, N, D, C, Q, E, G, H, I, L, K, M, F, P, S, T, W, Y, and V. Although protein sequences and DNA sequences belong to symbolic sequences, the methods for the graphical representation of protein sequences are relatively less popular, compared with DNA sequences. The key reason is that the extension of DNA graphical representation to protein sequences enormously increases the number of possible alternative assignments for these 20 amino acids. The amino acid sequence is the key to discover protein structure and function in the cell, so analysis of amino acid sequences is a very important part of postgenomic studies. The graphical representation study of protein sequences emerged very recently. The first visualization protein model was proposed by Randić et al. until 2004 [19]. Some researchers have studied on graphical representation of protein sequences from different perspectives [20–29].

In this paper, we introduce DV-curve graphical representation of protein sequences based on the detailed hydrophobic-hydrophilic (HP) model of amino acids. According to the important hydropathy, this approach is accompanied by a relatively small number of arbitrary choices associated with the graphical representation of proteins. Also, this representation has relatively good visualization effect to describe protein sequences in a perceivable way. As its application, we analyze the similarity/dissimilarity among some ND6 sequences and construct the phylogenetic tree of 35 coronavirus spike proteins.

## 2. DV-Curve Representation of Protein Sequences

### 2.1. Classification of Protein Sequences

The amino acid sequence is closely related to biological function. The closer the genetic relationship is, the smaller the difference in amino acid composition between them will be. Over the past thirty years, the characteristics of protein sequences have been studied by establishing different classified models [21–24, 26, 27]. A well-known model of protein sequences is the hydrophobic (H or nonpolar)-hydrophilic (P or polar), that is, the HP model may be too simple and lacks enough consideration on the heterogeneity and the complexity of the natural set of residues [30]. Based on Brown's work [31], 20 different kinds of amino acids are divided into four groups: nonpolar (np), negative polar (nep), uncharged polar (up), and positive polar (pp). This is called the detailed HP model, which can provide more information than the original HP model.

For a given protein sequence *S* = *S*_1_ *S*_2_ ⋯ *S*_*n*_ with length *n*, where *S*_*i*_ is the letter in the *i*th position among the protein sequence (*i* = 1,2,…, *n*), we define a primary protein sequence as a symbolic sequence which includes four letters according to the following rule:

(1) $$\begin{array}{c} b_{i}=\{\begin{array}{cc} B_{1},\; & \text{if}\;S_{i}\in \text{np},\\ B_{2},\; & \text{if}\;S_{i}\in \text{nep},\\ B_{3},\; & \text{if}\;S_{i}\in \text{up},\\ B_{4},\; & \text{if}\;S_{i}\in \text{pp}. \end{array} \end{array}$$

So *b*_*i*_ is the substitution for *S*_*i*_, and then we obtain a sequence *G*(*s*) = *b*_1_ *b*_2_ ⋯ *b*_*n*_. Here *b*_*i*_ is a letter of the alphabet *B*_1_, *B*_2_, *B*_3_, *B*_4_. For example, for a given protein primary sequence *S* = *WTFESRNDPAK*, we can transform it into a new sequence according to the above rule, *G*(*S*) = *B*_1_ *B*_3_ *B*_1_ *B*_2_ *B*_3_ *B*_4_ *B*_3_ *B*_2_ *B*_1_ *B*_1_ *B*_4_. Via comparison of the reduced sequence, it will be easier to understand the biological function of various kinds of amino acid residues.

### 2.2. Graphical Representation of Protein Sequences

**Figure 1:**
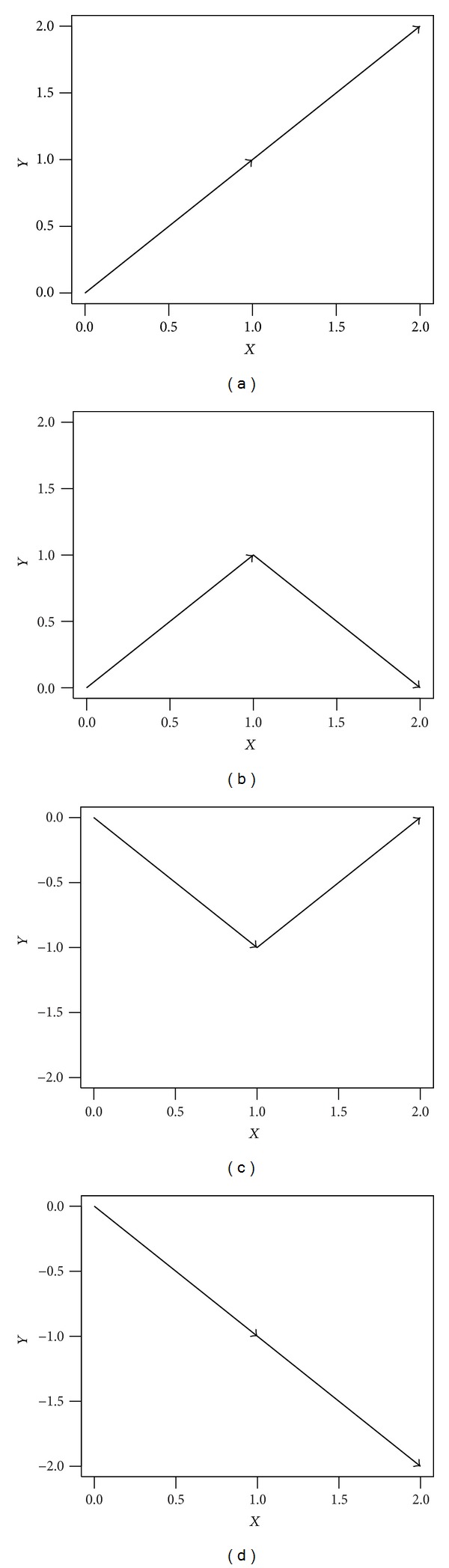
The representation of four alphabets of DV-curve: (a) *B*_1_, (b) *B*_2_, (c) *B*_3_, and (d) *B*_4_.

In this section, we will construct DV-curve representation of protein sequence. Given any protein primary sequence with length *n*, we can transform it into a new sequence composed of a character set of *B*_1_, *B*_2_, *B*_3_, *B*_4_. As shown in Figure 1, these alphabets are assigned, respectively, by consecutive vectors as follows:

(2) B1⟹(1,1),(1,1)B2⟹(1,1),(1,−1)B3⟹(1,−1),(1,1)B4⟹(1,−1),(1,−1).

**Figure 2:**
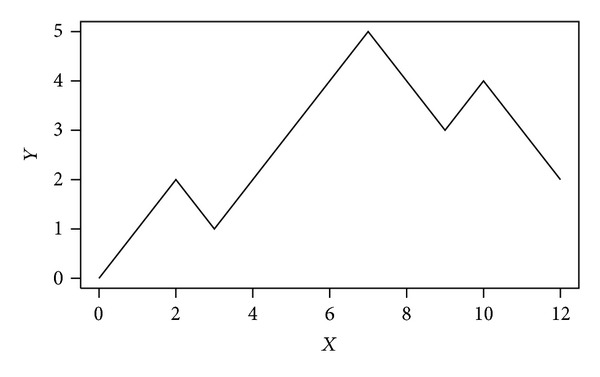
The DV-curve of sequence “WTFESR.”

We connect adjacent dots with lines and then obtain a dual-vector curve form. This process is shown in Figure 2.

Based on the construction of DV-curve, we obtain two mathematical models, respectively. One is “from protein sequence to DV-curve,” and the other is “from DV-curve to protein sequence.” Firstly, we give some common symbols and variables. (1) According to the classification rule, we describe a protein sequence as *G*(*S*) = *b*_1_ *b*_2_ *b*_3_ ⋯ *b*_*n*_, where *b*_*i*_ ∈ {*B*_1_, *B*_2_, *B*_3_, *B*_4_} with length *n*. It means that the protein sequence *S* is connected by these alphabets. (2)  (*x*_*i*_, *y*_*i*_) is the coordinate of the *i*th point of DV-curve, and (*x*_0_, *y*_0_) = (0,0) is the start point.

*Model One*. Given a primary protein sequence, we can draw its DV-curve:

(3) $$\begin{array}{c} x_{2i-1}=2i-1,\;i=1,2,\ldots ,n,\\ x_{2i}=2i,\;i=1,2,\ldots ,n,\\ y_{2i-1}=\{\begin{array}{cc} y_{2i-2}+1, & \text{if}\;b_{i}=B_{1}\;\text{or}\;B_{2},\\ y_{2i-2}-1, & \text{if}\;b_{i}=B_{3}\;\text{or}\;B_{4}, \end{array}\\ y_{2i}=\{\begin{array}{cc} y_{2i-1}+1, & \text{if}\;b_{i}=B_{1}\;\text{or}\;\;B_{3},\\ y_{2i-1}-1, & \text{if}\;b_{i}=B_{2}\;\text{or}\;\;B_{4}. \end{array} \end{array}$$

According to the above four formulas, the coordinate of each point (*x*_*i*_, *y*_*i*_) can be calculated. Then we connect all the points with beelines, and the DV-curve is obtained.

*Model Two*. Given a DV-curve, we can also obtain the coarse-grained description of the protein sequence based on the detailed HP-model:

(4) $$\begin{array}{c} G\left(S_{i}\right)=\{\begin{array}{ccc} B_{1}, & \text{if}\;y_{2i-1}-y_{2i-2}=1, & y_{2i}-y_{2i-1}=1,\\ B_{2}, & \text{if}\;y_{2i-1}-y_{2i-2}=1, & y_{2i}-y_{2i-1}=-1,\\ B_{3}, & \;\text{if}\;y_{2i-1}-y_{2i-2}=-1, & y_{2i}-y_{2i-1}=1,\\ B_{4}, & \;\text{if}\;y_{2i-1}-y_{2i-2}=-1, & y_{2i}-y_{2i-1}=-1. \end{array} \end{array}$$

Here *i* = 1,2, 3,…, *n*. If each point (*x*_*i*_, *y*_*i*_) of DV-curve is given in this model, we can get each *B*_*i*_ according to the above formulas. So the simplified protein sequence *G*(*S*) = *b*_1_ *b*_2_ ⋯ *b*_*n*_ can be recovered; here *b*_*i*_ ∈ {*B*_1_,  *B*_2_,  *B*_3_,  *B*_4_} with length *n*.

## 3. Numerical Characterization of Protein Sequences

In order to facilitate quantitative comparisons of sequences, we will give numerical characterization of graphical curve as the descriptor. In general, we transform the graphical representation into a mathematical object like a matrix in order to draw some invariants. The frequently used matrices include *E* matrix, *M* matrix, *L* matrix, and *L* ^*k*^ matrix proposed by Randić et al. [6, 8, 32–34]. Of course, there are some other matrix invariants such as the average matrix element, the average row sum, the Wiener number, and the ALE-index et al. These methods were used widely and proved to be useful. Here, we use the *CM*_*xy*_ as an alternative sequence invariant proposed by Liao et al. [35]:

(5) $$\begin{array}{c} \left(x_{c},y_{c}\right)=\left(\frac{1}{2n+1}\overset{2n}{\underset{i=0}{\sum }}x_{i},\;\;\frac{1}{2n+1}\overset{2n}{\underset{i=0}{\sum }}y_{i}\right),\\ CM_{xy}=\frac{1}{2n+1}\overset{2n}{\underset{i=0}{\sum }}\left(x_{i}-x_{c}\right)\left(y_{i}-y_{c}\right). \end{array}$$

Obviously, this index is relatively simple for calculation so that this index can provide some convenience for long sequences.

If we adjust the order of *B*_1_,  *B*_2_,  *B*_3_,  *B*_4_ corresponding to basic dual vectors, we can get another curve. So for a given sequence, we can get 4! = 24 different DV-curves totally. Therefore, a protein primary sequence can be characterized by a 24-component vector as follows: $\vec{v}=[CM1_{xy},CM2_{xy},\ldots ,CM24_{xy}]$. Based on the vectors, we can compare different protein sequences. Generally speaking, we can obtain the similarities of the two vectors by calculating Euclidean distance. If two sequences are similar, the distance between two corresponding points should be small. Given two species *i* and *j*, the corresponding vectors are $\vec{v_{i}}=[CMi1_{xy},CMi2_{xy},\ldots ,CMi24_{xy}]$ and $\vec{v_{j}}=[CMj1_{xy},CMj2_{xy},\ldots ,CMj24_{xy}]$, respectively; then we have $d(\vec{v_{i}},\vec{v_{j}})=\sqrt{\sum_{k=1}^{24}{\left(CMik_{xy}-CMjk_{xy}\right)}^{2}}$.

## 4. Application

The comparison on biology sequences is one of the most important parts in bioinformatics when analyzing similarities of function and properties. In this section, we will give two main applications of this new graphical representation. One is similarity analysis based on visual graphics. Generally, similarity analysis can be divided into two types of methodologies to conduct the comparison: sequence alignment and sequence descriptors comparison. When recognizing figures, our brain is more helpful for similarity analysis in multiple sequences. So it is desirable to propose similarity analysis by inspecting the DV-curve of protein. The other is evolutionary homology analysis based on the numerical characterization of DV-curve, and we construct a 24-component vector to characterize any protein sequence. As further work, the phylogenetic tree of 35 coronavirus spike proteins is constructed.

### 4.1. Similarity Analysis Based on Visual Inspection of the Protein DV-Curve Graphs

Since Smith and Waterman developed a dynamic programming algorithm in 1981, many alignment algorithms identifying whether two biological sequences are similar to each other have been studied. These methods are proved to be efficient. However, multiple sequence alignment (MSA) of several hundred sequences has always produced a bottleneck.

In 1994, MSA was proved to be an NP-complete problem by Wang and Jiang [36]. Moreover, most experts think that it is impossible until now to build a deterministic polynomial algorithm to handle an NP-complete problem. It needs to exhaust almost billions or trillions of years. Except long computational time, there also exists possible bias of multiple sequence alignments for multiple occurrences of highly similar sequence [37].

However, our brain is much more powerful than computer when recognizing different figures. So it can help us to analyze the similarity in multiple sequences. If we can provide a simple, intuitional, clear, and nondegenerate 2D graphical representation of protein sequences, molecular biologists may easily find out which sequence is most similar or dissimilar to the given target sequence. And next they can use alignment algorithms for further confirmation.

According to our proposed definition of protein DV-curve, we can draw the curves of some ND6 (NADH dehydrogenase subunit 6) proteins in order to conveniently compare them. Protein sequences that are used to prove our approach were downloaded from GenBank: human (*YP*_003024037.1), gorilla (*NP*_008223), chimpanzee (*NP*_008197), wallaroo (*NP*_007405), harbor seal (H. seal) (*NP*_006939), gray seal (G. seal) (*NP*_007080), rat (*AP*_004903), and mouse (*NP*_904339), and the same data set was used in [26, 27].

**Figure 3:**
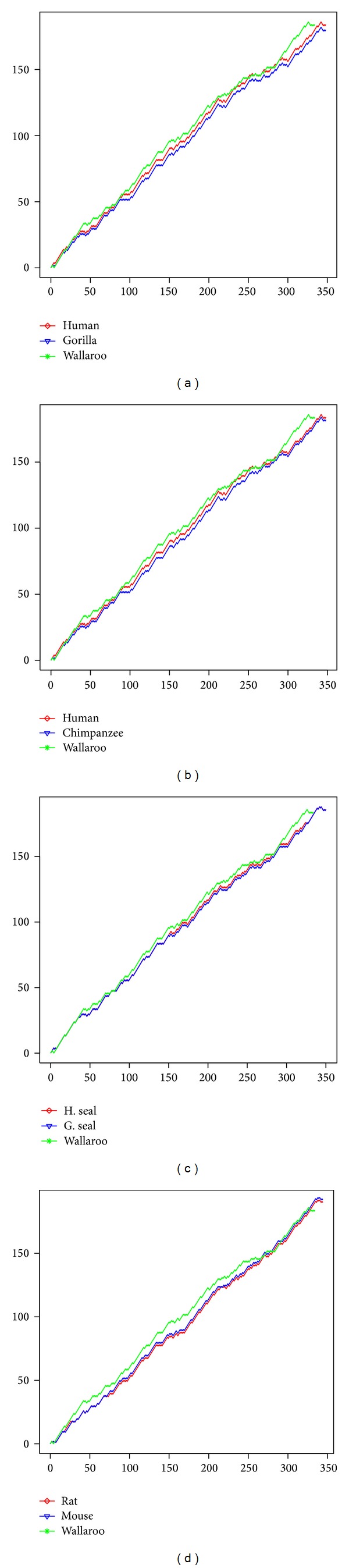
The DV-curve graphical representations of different ND6 proteins.

In Figure 3, it is evident that protein graph of wallaroo is obviously different from the other species because it is the most remote species from the remaining mammals. Furthermore, we can see human and chimpanzee have similar curves, harbor seal and gray seal's curves are almost identical, and two curves of rat and mouse are very similar. All these results not only are consistent with the conclusions drawn by Smith-Waterman algorithm, but also agree well with the known fact of evolution and results drawn by other authors [26, 27, 38–40]. In particular, compared with the conclusion of [27], the DV-curve representation reflecting the similarities of sequences is more simple, intuitional, and visible.

### 4.2. The Phylogenetic Analysis among the Spike Glycoprotein of Coronaviruses

Coronaviruses belong to order Nidovirales, family Coronaviridae, and genus* Coronavirus*. They are a diverse group of large, enveloped, single-stranded RNA viruses that cause respiratory and enteric diseases in humans and other animals. Generally, coronaviruses can be divided into three groups: the first group and the second group come from mammalian; the third group comes from poultry (chicken and turkey). A novel coronavirus has been identified as the cause of the outbreak of severe acute respiratory syndrome (SARS). Previous phylogenetic analysis based on sequence alignments shows that SARS-CoVs come from a new group distantly related to the above three groups of previously characterized coronaviruses [41, 42]. The spike (S) protein, which is common to all known coronaviruses, is crucial for viral attachment and entry into the host cell. To illustrate the use of DV-curve of protein sequences, we will construct the phylogenetic tree of 35 coronavirus spike proteins of Table 1.

As we have described above, a protein sequence can be associated with a 24-component vector. Given two species *i* and *j*, we can calculate the distance between them. Our datasets used in this paper were downloaded from GenBank (see Table 1 for details). Corresponding to 35 spike proteins, a 35 × 35 real symmetric matrix *D* = (*d*_*ij*_) is obtained and used to reflect the evolutionary distance of them. Using the UPGMA program included in PHYLIP package 3.65, we can construct the phylogenetic tree of these 35 species [43, 44]. The branch lengths are not scaled according to the distances and only the topology of the tree is concerned.

**Figure 4:**
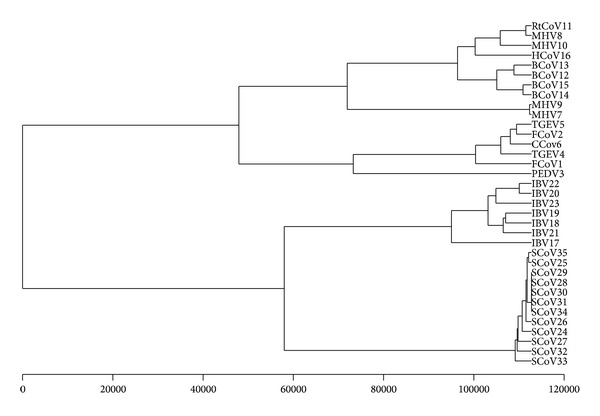
The phylogenetic tree based on the spike proteins.

Figure 4 shows coronaviruses can be overall divided into four groups. Furthermore, it is evident that SARS-CoVs appear to cluster together and form a separate branch, which can be distinguished easily from the other three groups of coronaviruses.

RtCoV11, MHV8, MHV10, HCoV16, BCoV13, BCoV12, BCoV15, BCoV14, MHV9, and MHV7, which belong to group 2, are situated at an independent branch, while TGEV5, FCoV2, CCoV6, TGEV4, FCoV1, and PEDV3, belonging to group 1, tend to cluster together. Meanwhile, the group 3 coronaviruses, including IBV22, IBV20, IBV23, IBV19, IBV18, IBV21, and IBV17, tend to cluster together in another branch. The resulting monophyletic clusters agree well with the established taxonomic groups [45, 46]. The conclusion is similar to that reported by other authors [23, 24]. Compared with result [24], it is noteworthy that a closer look at the subtree of the first branch shows coronavirus from three different species; that is, MHV, BCoV, and HCoV can be separated clearly, while they cluster together in a subtree by Li's method. Obviously, our conclusion is more consistent with the known evolution fact.

## 5. Conclusion

According to the detailed hydrophobic-hydrophilic (HP) model of amino acids, we can reduce a protein primary sequence containing 20 amino acids into a four-letter sequence, which can be treated as a coarse-grained description of the protein primary sequence. Here we cannot avoid losing some information in the reduced sequences, but we can focus our main attention on the part of our interest.

Some alignment-free methods to analyze DNA sequences have been proposed. However, there are few alignment-free methods to analyze protein sequences. Our method realizes the generalization from DNA graphical representations to those of proteins acceptable and can be seen a valid supplement to graphical representation of protein sequences. Meanwhile we first propose to combine DV-curve and the detailed HP model together to describe protein sequences.

Compared with classical Smith-Waterman algorithm, the similarity/dissimilarity analysis results are consistent with DV-curve. In addition, the advantage of our method is that it can visualize the local and global features among different proteins no matter how long these sequences are and avoid degeneracy at the same time. The new approach is applied in two aspects: one is similarity intuitive analysis of ND6 protein sequences of several species and the other is phylogenetic analysis among 35 coronaviruses based on their spike proteins. Results have shown that our proposed method is more intuitional, simple, effectual, and feasible.

## Figures and Tables

**Table 1:** The information of 35 coronavirus spike proteins.

Number	Accession number	Abbreviation notation	Length (aa)	Group
1	P10033	FCoV1	1452	I
2	Q66928	FCoV2	1454	I
3	Q91AV1	PEDV3	1383	I
4	Q9DY22	TGEV4	1449	I
5	P18450	TGEV5	1449	I
6	P36300	CCoV6	1451	I
7	Q9J3E7	MHV7	1324	II
8	Q83331	MHV8	1361	II
9	P11224	MHV9	1324	II
10	O55253	MHV10	1360	II
11	Q9IKD1	RtCoV11	1360	II
12	P25190	BCoV12	1363	II
13	P15777	BCoV13	1363	II
14	Q9QAR5	BCoV14	1363	II
15	P36334	BCoV15	1363	II
16	P36334	HCoV16	1353	II
17	Q82666	IBV17	1166	III
18	P05135	IBV18	1163	III
19	P12722	IBV19	1154	III
20	Q64930	IBV20	1168	III
21	Q82624	IBV21	1159	III
22	P11223	IBV22	1162	III
23	Q98Y27	IBV23	1162	III
24	AAP41037	SCoV24	1255	IV
25	AAP300030	SCoV25	1255	IV
26	AAR91586	SCoV26	1255	IV
27	AAP51227	SCoV27	1255	IV
28	AAP33697	SCoV28	1255	IV
29	AAP13441	SCoV29	1255	IV
30	AAQ01597	SCoV30	1255	IV
31	AAU81608	SCoV31	1255	IV
32	AAS00003	SCoV32	1255	IV
33	AAR86788	SCoV33	1255	IV
34	AAR23250	SCoV34	1255	IV
35	AAT76147	SCoV35	1255	IV
